# Point-of-Care Ultrasound Diagnosis of Right Ventricular Rupture Post Cardiac Arrest After Thrombolysis in Myocardial Infarction

**DOI:** 10.5811/cpcem.2019.1.40664

**Published:** 2019-01-28

**Authors:** Jonathan Miller, Lorna Swarbrick, Butheyna Abdelhameed, Thomas Carter

**Affiliations:** * Palmerston North Hospital, Department of Emergency Medicine, Roslyn, Palmerston North, New Zealand; † University of Otago, Department of Surgery and Anesthesia, Dunedin, New Zealand; ‡ Ohio University Heritage College of Osteopathic Medicine, Department of Emergency Medicine, Athens, Ohio

## CASE PRESENTATION

An 81-year-old male was referred by his general practitioner with a troponin-T of 153 nanograms per liter (ng/L) (reference range <5 ng/L) and chest pain ongoing for 13 hours on arrival. Initial electrocardiogram showed 7-millimeter anterior ST elevation in leads V2–5. The case was discussed with cardiology at the nearest tertiary care center and plans were arranged for the patient’s transfer for percutaneous coronary intervention. Thrombolysis was withheld due to a known abdominal aortic aneurysm and a suspicious renal mass under investigation. While awaiting transfer, the patient suffered a ventricular tachycardia arrest, and cardiopulmonary resuscitation (CPR) was commenced. Point-of-care echocardiogram was performed, showing a hypokinetic myocardium. After four rounds of CPR, thrombolysis was given as a last resort. Repeat point-of-care echocardiography demonstrated irrecoverable injury; therefore, CPR was discontinued (Video).

## DISCUSSION

Right ventricle (RV) free wall rupture is a dangerous complication of myocardial infarction (MI) with a high mortality rate. Overall, cardiac rupture complicates approximately 5% of cases of acute MI,^1^ with left ventricular rupture accounting for the majority. RV rupture is comparatively rare and its identification via echocardiography rarer still. Over 10% of cases of free wall rupture occur in patients who subsequently died from ST-segment elevation myocardial infarction (STEMI).^2^ This case was likely a type I rupture: an abrupt, slit-like tear occurring in acute infarcts of less than 24 hours duration.^3^ Type II ruptures occur where the infarcted myocardium erodes before rupture and is covered by thrombus. Type III represents the perforation of a previously formed aneurysm.^3^ Examination and history findings are non-specific, but ultrasound for diagnosing ventricular rupture is greater than or equal to 70% sensitive and 90% specific.^4^

It has previously been shown that thrombolytic therapy is independently associated with increased incidence of cardiac rupture and this risk is elevated with prolonged time to administration of thrombolysis.^5^–^7^ Other risk factors include advanced age, female sex, previous cerebrovascular disease, chronic kidney disease, and congestive heart failure.^8^–^9^ Our patient fulfilled only one of these risk criteria. This case demonstrates the value of echocardiography in the diagnosis of RV free wall rupture, along with the risks of thrombolysis and the need for further research around RV rupture post-STEMI.

**CPC-EM Capsule**

What do we already know about this clinical entity?

*Most literature describing ventricular rupture discusses the left ventricle or the septum. There is minimal literature describing right ventricular free wall rupture*.

What is the major impact of the image(s)?

*Emergency clinicians may be faced with this rare complication and be able to direct treatment quickly with point-of-care ultrasound images*.

How might this improve emergency medicine practice?

*The treating group of clinicians felt it may be difficult to interpret a free wall rupture and act appropriately if it was not quickly identified*.

## Supplementary Information

Video Point-of-care echocardiogram demonstrating right ventricle free wall rupture.
